# Apelin inhibited epithelial−mesenchymal transition of podocytes in diabetic mice through downregulating immunoproteasome subunits β5i

**DOI:** 10.1038/s41419-018-1098-4

**Published:** 2018-10-09

**Authors:** Jiming Yin, Yangjia Wang, Jing Chang, Bin Li, Jia Zhang, Yu Liu, Song Lai, Ying Jiang, Huihua Li, Xiangjun Zeng

**Affiliations:** 1 Beijing You An Hospital, Capital Medical University, 100069 Beijing, China; 2 Beijing Institute of Hepatology, 100069 Beijing, China; 3 Department of Pathology and Pathophysiology, Capital Medical University, 100069 Beijing, China; 4 Department of Cardiology. Institute of Cardiovascular Diseases, First Affiliated Hospital of Dalian Medical University, No.193, Lianhe Road, Xigang District, 116011 Dalian, China

## Abstract

The epithelial−mesenchymal transition (EMT) of podocytes had been reported to be involved in the glomerular fibrosis in diabetic kidney diseases, which was regulated by TGFβ and NFκB pathways. And apelin, an adipokine which is upregulated in diabetic kidney diseases, was reported to be negatively correlated to TGFβ in polycystic kidney disease and attenuate EMT in renal tubular cells. Therefore, it is hypothesized that apelin might inhibit the EMT of podocytes through downregulating the expression and activation of TGFβ/Smad pathway in diabetic kidney diseases. The results showed that apelin in glomeruli of diabetic mice were increased and exogenous apelin inhibited the EMT of podocytes in diabetic mice, which were accompanied with the decreased expression of proteasome subunits β5i. The results from β5iKO mice confirmed that the inhibiting effects of apelin on EMT of podocytes in diabetic mice were dependent on β5i. The results from culture podocytes showed that apelin decreased the degradation of pIκB and promoted the translocation of IκB into nucleus through decreasing the expression of β5i, which would inhibit the promoting effects of NFκB on expression of TGFβ and followed by decreased activation of Smad pathway and EMT in podocytes. In conclusion, apelin might act as an EMT suppressor for podocytes to decrease the process of glomerular fibrosis in diabetic mice.

## Introduction

Emerging evidence indicated that podocytes would go epithelial−mesenchymal transition (EMT) in diabetic conditions by losing expression of highly specialized markers of podocyte such as nephrin, synapotodin, zonula occludens-1 (ZO-1), and Wilms’ tumor 1 (WT 1) and acquiring expression of new mesenchymal markers such as α-Smooth Muscle Actin (α-SMA), type I collagen (Col-I), type IV collagen (Col-IV), and fibronectin (FN)^1–3^. EMT of podocytes would result in glomerular fibrosis in diabetic kidney diseases (DKD)^4^. As DKD is the major cause of end-stage renal disease (ESRD), alleviating EMT of podocytes in DKD might prevent the progression of ESRD in patients with DKD.

Previous studies indicated that Apelin, an adipokine which is upregulated in DKD, was negatively correlated to renal fibrosis and TGFβ in polycystic kidney disease^5^. Meanwhile, apelin was reported to attenuate EMT in renal tubular cells^6^. Then, is apelin capable of inhibiting glomerular fibrosis induced by diabetes mellitus through reducing EMT of podocytes?

Among the factors inducing EMT, TGFβ was considered to be a key factor that was reported to be regulated by proteasomes and NFκB^7–12^. And apelin was reported to inhibit expression and activities of proteasomes in podocytes^12^. At the same time, proteasome was reported to regulate the protranscriptional activities of NFκB in macrophages^13,14^. Then is it possible that apelin may attenuate the EMT of podocytes through inhibiting proteasome which would regulate the activity of NFκB pathway by degrading IκB? The aim of this study was to demonstrate the effects and molecular mechanisms of apelin on EMT of podocytes in diabetic mice.

## Results

### Expression of apelin in glomeruli

**Fig. 1:**
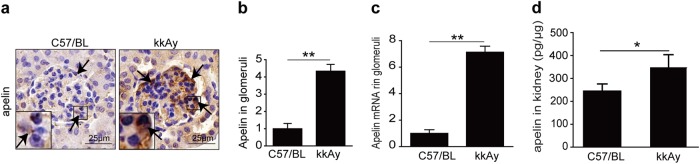
Expression of apelin in glomeruli. **a** Representative immunostaining of apelin in kidney sections from control and kkAy mice, as quantified in (**b**) using unpaired Student’s *t*-test (*n* = 6 mice per group, **p < 0.01). Scale bars represent 25 μm. **c** Apelin mRNA expression in the glomeruli of C57 and kkAy mice. *GAPDH* served as reference gene. Values represent the mean ± SD (*n* = 3 mice per group, **p < 0.01). **d** Apelin concentration in extraction of kidney detected with ELISA. Values represent the mean ± SD (*n* = 6 mice per group, *p < 0.05)

To evaluate whether the expression of apelin in glomeruli of diabetic mice was increased or not, immunostaining, qPCR, and ELISA were used. The results indicated that apelin was increased to 4.3 folds in glomeruli of type 2 diabetic mice (kkAy mice) as shown in Fig. 1a, b, apelin mRNA was increased to 7.1 folds in glomeruli of kkAy mice as shown in Fig. 1c, apelin concentration in homogenate was increased from 255 pg/μg to 362 pg/μg in glomeruli of kkAy mice as shown in Fig. 1d. These results confirmed that apelin was increased in glomeruli of diabetic mice, whose effects on glomerular fibrosis in diabetic mice were not determined.

### Effects of apelin on morphology of glomeruli

**Fig. 2:**
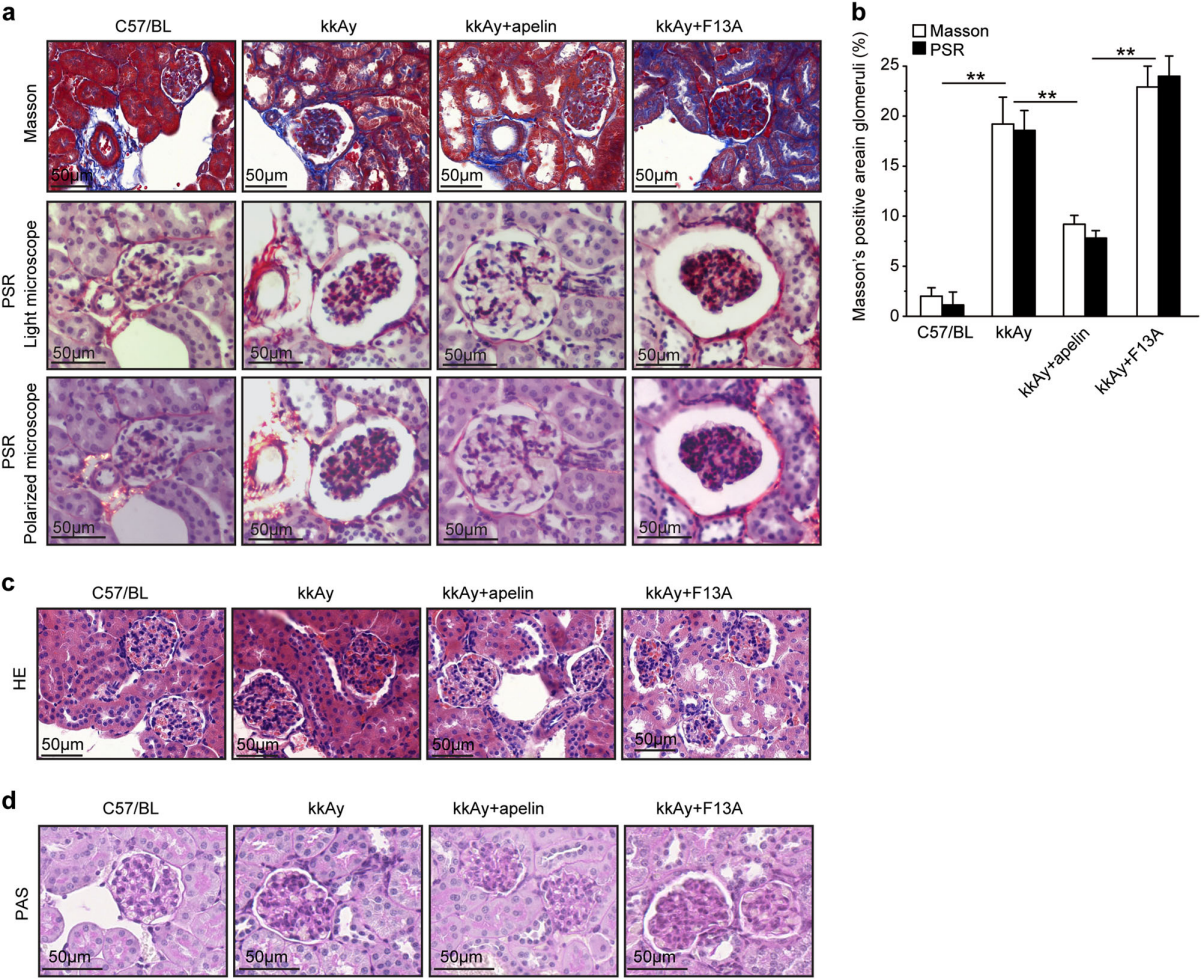
Apelin acted as a suppressor for glomerular fibrosis in diabetic mice. **a** Representative Masson Trichrome and Pico Sirius Red (PSR) staining in kidney sections from C57 and diabetic mice with or without apelin/F13A treatment as quantified in (**b**) using unpaired Student’s *t*-test (*n* = 6 mice per group, **p < 0.01). Scale bars represent 50 μm. **c** Representative H&E histology in kidney sections from C57 and diabetic mice with or without apelin/F13A treatment. Scale bars represent 50 μm. **d** Representative periodic acid Schiff (PAS) staining in kidney sections from C57 and diabetic mice with or without apelin/F13A treatment. Scale bars represent 50 μm

Masson staining showed that the collagen fibers that were stained as blue were increased in glomeruli of kkAy mice. Apelin decreased the blue-colored collagen fibers from 18.9 to 8.7% in glomeruli of kkAy mice, while F13A increased the blue-colored collagen fibers to 6.9% in glomeruli (Fig. 2a).

The results from PSR staining showed that the red-colored collagen was increased in glomeruli of kkAy mice compared to that of C57/BL (17.1 ± 1.6% and 2.1 ± 1.3%, *p* < 0.05, *n* = 6). Apelin decreased the red-colored collagen in glomeruli of kkAy mice to 6.9%, while F13A increased the red-colored collagen to 23.0% in glomeruli of kkAy mice. However, observing with polarized light microscope did not get refractive orange light (collagen I) or refractive green light (collagen III) in glomeruli, which might be due to the low sensitivity of PSR staining or no collagen I and III were deposited in the glomeruli (Fig. 2a).

HE staining showed that the number of cells in glomeruli was increased in kkAy mice. Apelin decreased the number of cells in glomeruli of kkAy mice, while F13A increased the number of cells in glomeruli of kkAy mice (Fig. 2b).

PAS staining showed that matrix deposition in glomeruli was increased in kkAy mice. Apelin decreased the matrix deposition in glomeruli of kkAy mice, while F13A increased the matrix deposition in glomeruli of kkAy mice (Fig. 2c).

### Effects of apelin on EMT of podocytes in glomeruli

**Fig. 3:**
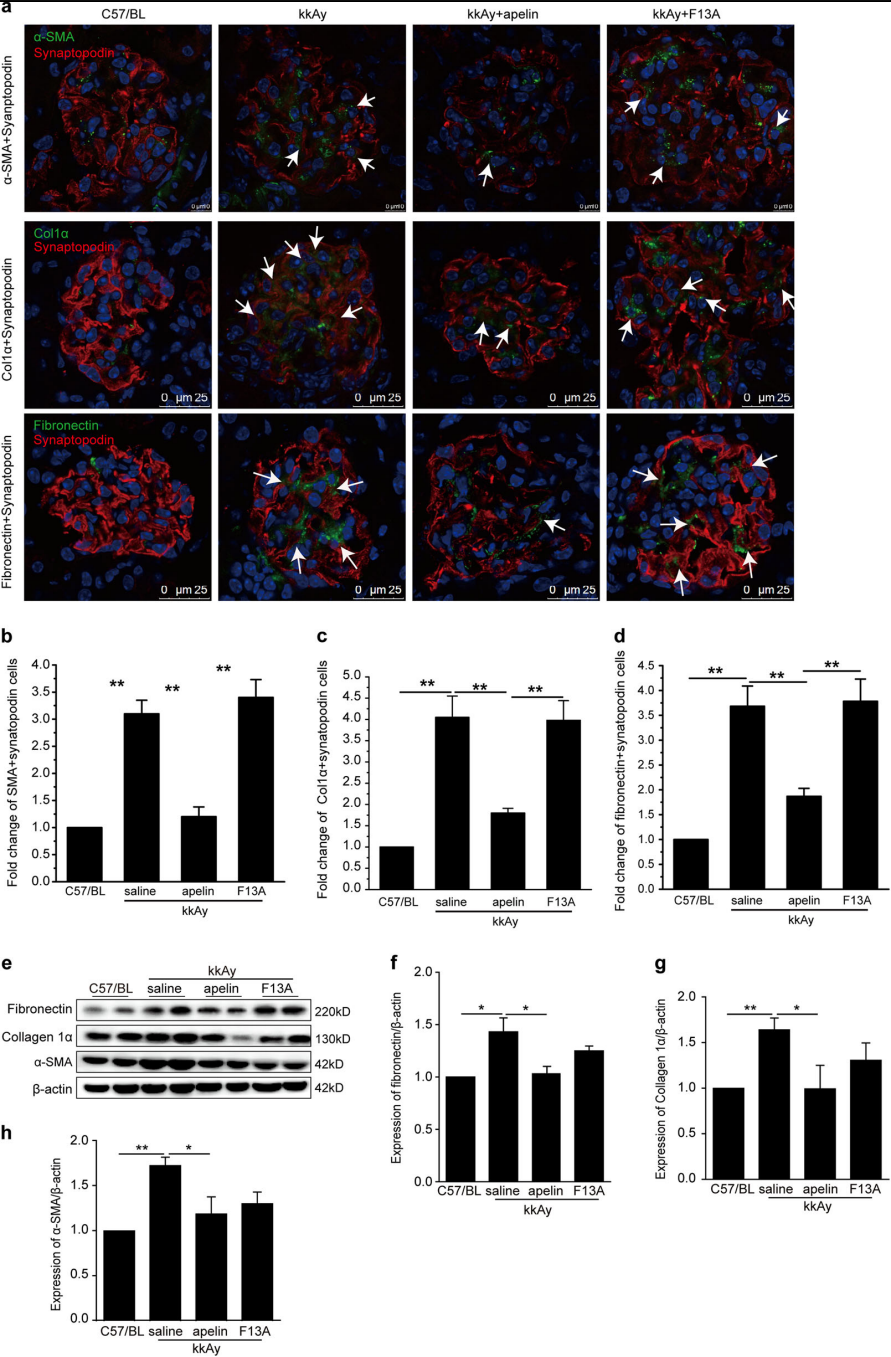
Apelin suppressed the expression of markers for epithelial to mesenchymal transformation (EMT) in podocytes. **a** Representative staining for α-SMA and synaptopodin as quantified in (**b**), staining for collagen 1α (Col1α) and synaptopodin as quantified in (**c**) and staining for fibronectin as quantified in (**d**). Scale bars represent 10 or 25 μm as shown in the photos. White arrows showed the positive podocytes for EMT markers. **e** Representative images of western blot for EMT markers in glomeruli. **f**–**h** Apelin inhibited the increased expression of α-SMA, fibronectin, and collagen 1α in glomeruli of kkAy mice. Values represent the mean ± SD (*n* = 6 mice per group, *p < 0.05 **p < 0.01)

To verify the effects of apelin on EMT of podocytes in diabetic mice, EMT markers (α-SMA, collagen1α, and fibronectin) were stained with synapotopodin at the same time. The cells positively stained with both synapotopodin and EMT markers were considered as EMT cells. The results indicated that EMT cells were increased to 3.0~4.0 folds in kkAy mice compared to C57/BL mice. Apelin reversed these cells back to 1.2~2.0 folds in kkAy mice, while F13A, the analog of apelin, did not change the EMT cells in glomeruli of kkAy mice (Fig. 3a–d). These results suggested that apelin inhibit the process of EMT in podocytes in diabetic mice.

To confirm the expression of EMT markers in glomeruli, western blot was used to detect the protein levels of α-SMA, collagen1α, and fibronectin in glomeruli. The results showed that protein levels of α-SMA, collagen1α, and fibronectin were increased to 1.4–1.8 folds in glomeruli of diabetic mice compared to that of control (*p* < 0.05, *n* = 3), which were reversed by apelin back to almost normal (*p* < 0.05, *n* = 3, Fig. 3e–h). Meanwhile, F13A did not show significant effects on these proteins in glomeruli. These results confirmed that apelin inhibit the expression of EMT in glomeruli of diabetic mice.

**Fig. 4:**
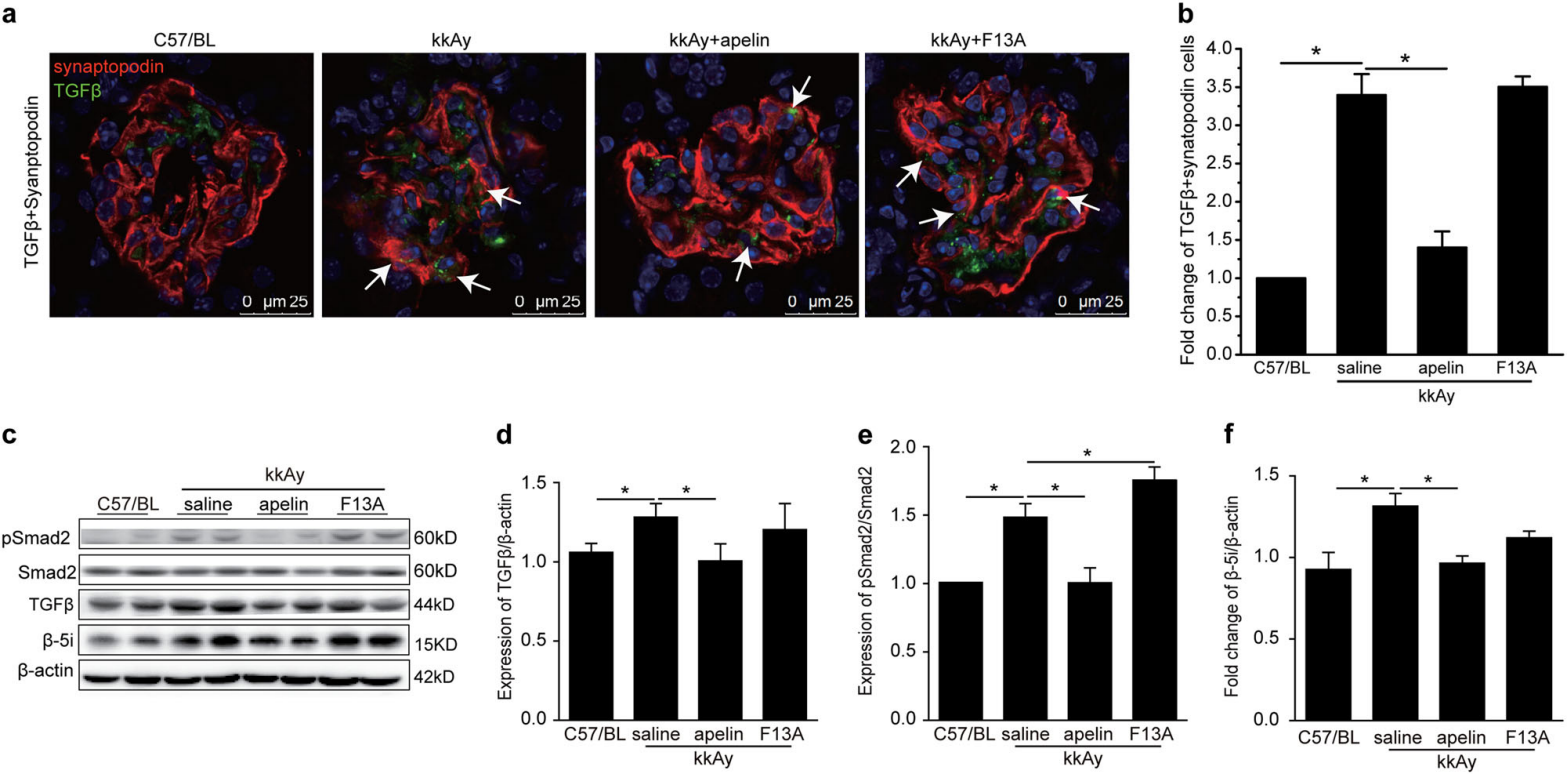
Apelin suppressed expression of TGFβ in glomeruli. **a** Representative staining for TGFβ and synaptopodin in kidney sections from C57 and diabetic mice with or without apelin/F13A treatment as quantified in (**b**) using unpaired Student’s *t* test. **c** Representative images of western blot for TGFβ/Smad pathway and β5i in glomeruli. **d**–**f** Apelin inhibited the TGFβ/Smad pathway and β5i in glomeruli of kkAy mice, while F13A enhanced the activation and expression of these pathways. Values represent the mean ± SD (*n* = 6 mice per group, *p < 0.01). Scale bars represent 50 μm

TGFβ was reported to be involved in the process of EMT; therefore, TGFβ and synapotopodin were stained to explore the effects of apelin on EMT of podocytes. The results indicated that both TGFβ- and synapotopodin-positive cells were increased to 3.7 folds in glomeruli of kkAy mice compared to C57/BL mice. Apelin decreased both TGFβ- and synapotopodin-positive cells to 1.47 folds in glomeruli of kkAy mice, while F13A did not show any effects on TGFβ in glomeruli of kkAy mice (*p* < 0.01, *n* = 3, Fig. 4a, b).

To confirm the effects of apelin on TGFβ pathway in glomeruli, western blot was used to detect the protein levels of TGFβ and Smad2/3. The results showed that TGFβ was increased to 1.3 folds of control in diabetic mice, which was reversed to 0.9 folds by apelin in glomeruli of mice (*p* < 0.05, *n* = 3, Fig. 4c, d). As the downstream of TGFβ, Smad was also detected with western blot. The results showed that pSmad was increased to 1.5 folds of control in glomeruli of diabetic mice and reversed by apelin to 1.02 folds, while F13A increased pSmad to 1.73 folds in diabetic mice (*p* < 0.05, *n* = 3, Fig. 4c–e). Interestingly, β5i, one of the proteasome subunit, displayed the same trend as TGFβ/Smad pathway in the glomeruli (*p* < 0.05, *n* = 3, Fig. 4c–e). These results suggested that inactivation TGFβ/Smad pathway induced by apelin might be correlated to β5i in glomeruli of diabetic mice.

### Effects of apelin on α-SMA, collagen I and fibronectin in high-glucose-treated podocytes

**Fig. 5:**
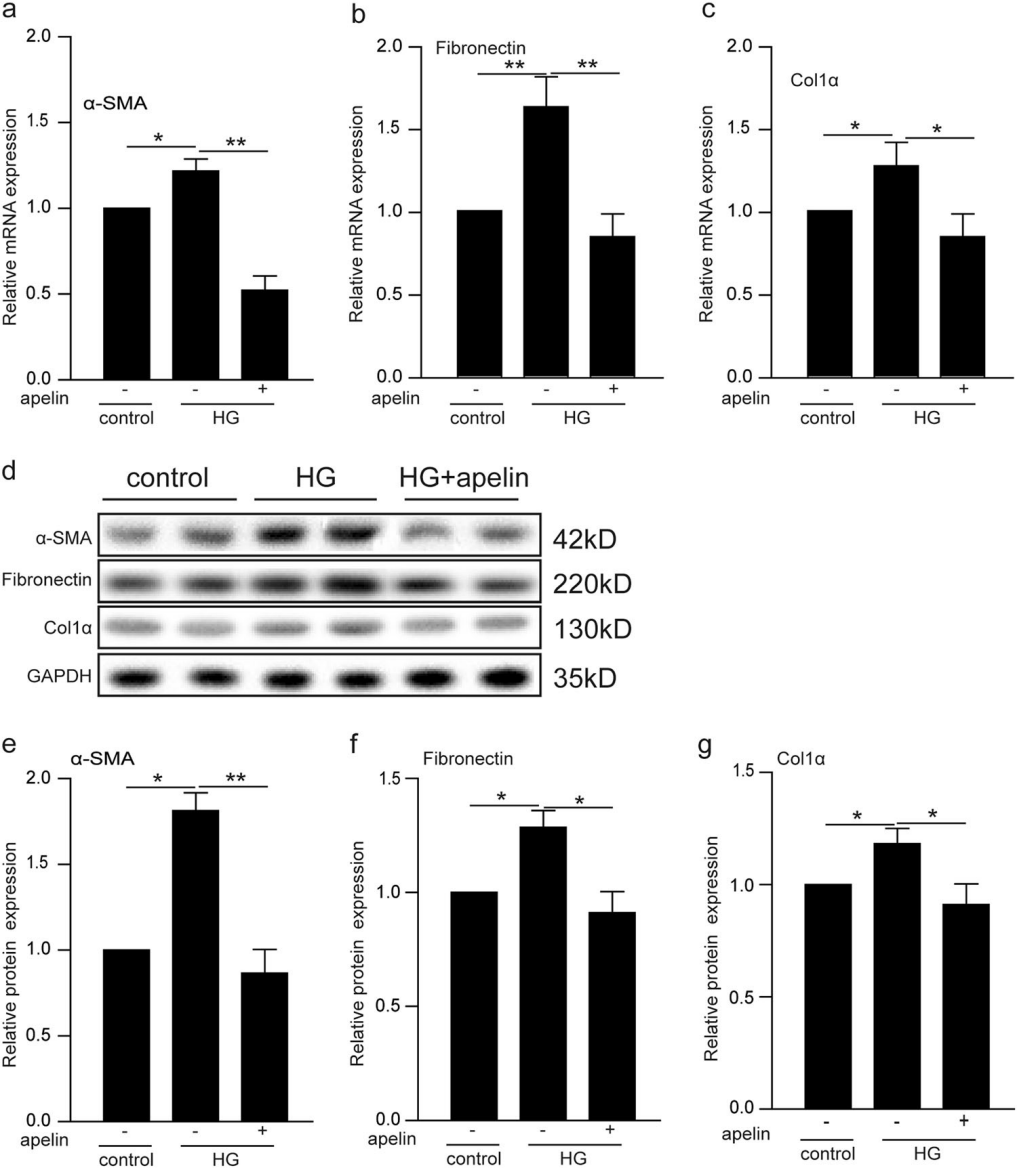
Apelin suppressed expression of EMT markers in high-glucose-treated podocytes. **a**–**c** High-glucose (HG) treatment increased α-SMA, fibronectin, and collagen1α in podocytes, while apelin reversed the expression of α-SMA, fibronectin, and collagen 1α in high-glucose-treated podocytes. **d** Representative images of western blot for EMT markers in podocytes. **e**–**g** Apelin inhibited the increased expression of α-SMA, fibronectin, and collagen 1α in high-glucose-treated podocytes. Values represent the mean ± SD (*n* = 3, *p < 0.05,**p < 0.01)

To evaluate the effects of apelin on EMT of podocytes induced by high glucose, PCR and western blot were used to detect the mRNA and protein of EMT markers (α-SMA, collagen1α, and fibronectin). The results showed that transcriptions of α-SMA, fibronectin, and collagen1α mRNAs were induced by high glucose to 1.2, 1.7, and 1.3 folds in podocytes compared to the control group, while apelin reversed these transcriptions to 0.5–0.7 folds in podocytes compared to the control group (Fig. 5a–c). Meanwhile, results of western blot showed that expression of α-SMA, fibronectin, and collagen1α were increased to 1.75, 1.35 and 1.2 folds in podocytes compared to the control group, while apelin reversed these expression to 0.8–0.9 folds in podocytes compared to the control group (Fig. 5e–h).

### Immunoproteasome β5i and NFκB involved in the effects of apelin on EMT of podocytes

**Fig. 6:**
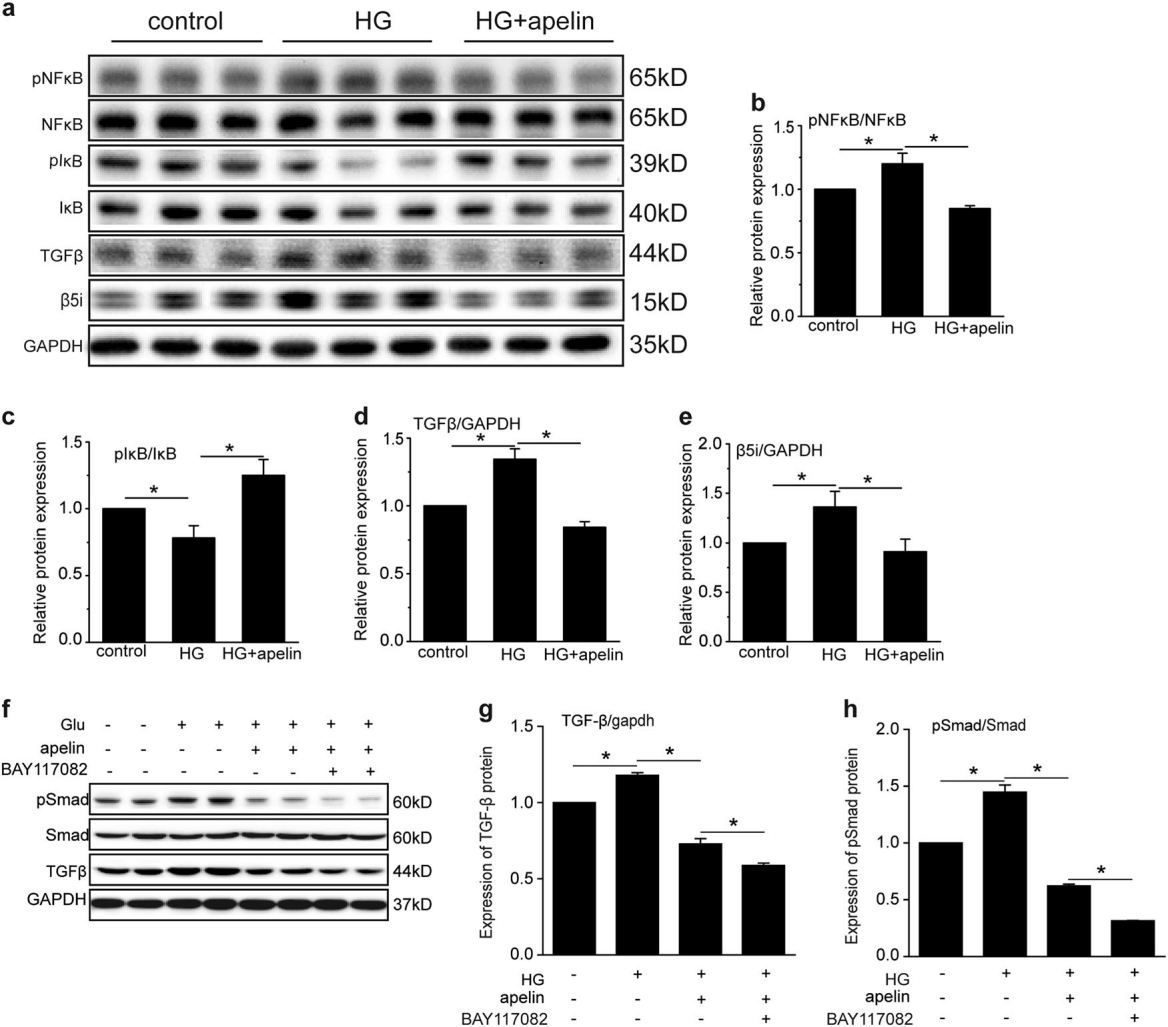
Apelin suppressed cell signaling pathways mediating EMT. **a** Representative images of western blot for cell signaling pathway in podocytes with or without apelin or high-glucose (HG) treatment. **b** Apelin inhibited the phosphorylation of NFκB in high-glucose-treated podocytes. **c** Apelin increased the phosphorylation of IκB in high-glucose-treated podocytes. **d** Apelin inhibited the expression of TGFβ in high-glucose-treated podocytes. **e** Apelin inhibited the expression of proteasome subunit β5i in high-glucose-treated podocytes. **f** Representative images of western blot for TGFβ/Smad pathway after BAY117082 and apelin treatment in podocytes. **g**, **h** Quantification of TGFβ/Smad pathway in (**f**). Values represent the mean ± SD (*n* = 3, *p < 0.05)

To verify the cell signaling pathways involved in the effects of apelin on EMT of podocytes in diabetic mice, TGFβ pathway was detected with western blot and qPCR in high-glucose and apelin-treated podocytes. The results showed that high glucose induced the transcription and expression of TGFβ in podocytes to 1.15 and 1.37 folds of control cells, and apelin reversed that of TGFβ to 0.4 and 0.8 folds (Fig. 6a–f). Meanwhile, NFκB, which was reported to mediate the transcription of TGFβ, was phosphorylated to be 1.2 folds of control cells by high glucose, and apelin reversed the pNFκB back to 0.8 folds of control cells in high-glucose-treated podocytes (Fig. 6a, b). To demonstrate the effects of NFκB on transcription of TGFβ, BAY117082 was used to inhibit the activation of NFκB. The results showed that expression of TGFβ was down-regulated to 0.6 folds with high glucose, apelin, and BAY117082 treatment compared to 0.8 folds in high-glucose- and apelin-treated podocytes (*p* < 0.05, *n* = 3, Fig. 6g, h). Meanwhile, phosphorylation of Smad was decreased too after BAY117082 was applied (*p* < 0.05, *n* = 3, Fig. 6g–i). These results suggested that high-glucose-induced TGFβ expression might be due to the phosphorylation of NFκB which would increase the translocation of NFκB into nucleus and promote the transcription and expression of TGFβ.

However, IκB, which is the repressor of NFκB, was phosphorylated by high glucose to 0.76 folds of control, and apelin increased pIκB to 1.25 folds of control in high-glucose-treated podocytes (*p* < 0.05, *n* = 3, Fig. 6a–c). These results are not consistent with that of NFκB and TGFβ, because increased pIκB would release more NFκB to promote TGFβ expression, but TGFβ was not increased with increased pIκB.

It is reported that pIκB might translocate into nucleus to inhibit transcription of TGFβ due to the decreased degradation of pIκB with proteasomes^13,14^; therefore, proteasome subunit β5i were detected. The results showed that high-glucose treatment increased the expression of proteasome subunit β5i to 1.4 folds of control in podocytes, which was reversed by apelin to 0.9 folds (*p* < 0.05, *n* = 3, Fig. 6g, e). These results suggested that apelin-inhibited EMT of podocytes in diabetic mice might be due to the inhibition of proteasome subunit β5i.

### Effects of immunoproteasome β5i knockout on EMT of podocytes in glomeruli

To evaluate the effects of β5i on EMT of podocytes with or without apelin treatment, immunoproteasome β5i knockout mice were adopted to repeat diabetic model with or without apelin treatment. The results showed that STZ injection successfully increased blood glucose both in wild-type mice and β5i knockout mice, and apelin treatment did not show significant influence on blood glucose of STZ-treated mice (Supplementary Figure 1).

**Fig. 7:**
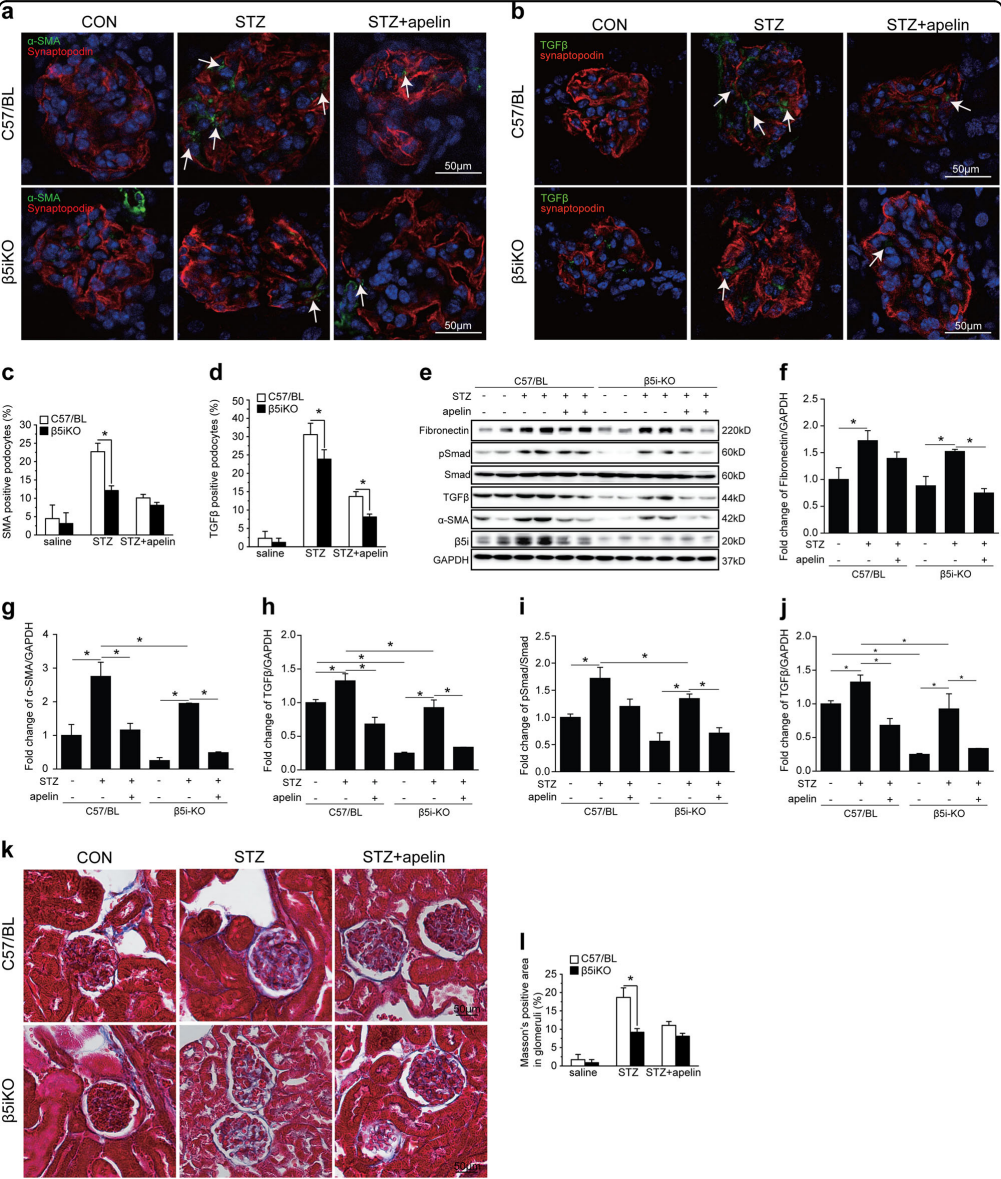
Proteasome subunit β5i knockout enhanced the inhibiting effects of apelin on EMT of podocytes in glomeruli of diabetic mice. **a** Representative staining for α-SMA and synaptopodin in STZ-treated C57 and β5i knockout (β5iKO) mice with or without apelin treatment as quantified in (**c**) using unpaired Student’s *t* test (*n* = 6 mice per group, *p < 0.05). Scale bars represent 50 μm. **b** Representative staining for TGFβ and synaptopodin in STZ-treated C57 and β5i knockout (β5iKO) mice with or without apelin treatment as quantified in (**d**) using unpaired Student’s *t* test (*n* = 6 mice per group, *p < 0.05). Scale bars represent 50 μm. **e** Representative images of western blot for EMT markers and TGF/Smad pathway in glomeruli. **f**–**j** Quantification of EMT markers and TGF/Smad pathway in (**e**). **k** Representative Masson staining in kidney sections from STZ-treated C57 and β5i knockout (β5iKO) mice with or without apelin treatment as quantified in (**l**) using unpaired Student’s *t* test (*n* = 6 mice per group, *p < 0.05). Scale bars represent 50 μm

Masson staining results showed that fibrosis area was increased from 2.3 to 17.8% in glomeruli of STZ-treated mice and decreased to 11.1% in glomeruli of STZ and apelin-treated C57/BL mice. Meanwhile, β5i knockout significantly decreased fibrosis area in glomeruli of STZ-treated mice to 9.7%, but did not show significant influence on reversing effects of apelin on glomerular fibrosis in diabetic mice (7.8% in apelin and STZ-treated β5i knockout mice as shown in Fig. 7k, i).

SMA and synapotopodin staining results showed that STZ increased SMA-positive podocytes (EMT of podocytes) from 4.9 to 22.1%, which was reversed by apelin to 10.2% in glomeruli of C57/BL mice. And β5i knockout significantly decreased SMA-positive podocytes in STZ-treated mice to 12.4%, but did not show significant influence on reversing effects of apelin on EMT of podocytes (7.9% in apelin and STZ-treated β5i knockout mice as shown in Fig. 7a–c).

TGFβ and synapotopodin staining showed that STZ increased TGFβ-positive podocytes from 2.5 to 31.2%, which was reversed by apelin to 13.1% of C57/BL mice. And β5i knockout significantly decreased SMA-positive podocytes in glomeruli of STZ-treated mice with or without apelin treatment (23.1 and 7.5% separately in glomeruli as shown in Fig. 7b–d).

To confirm the effects and cell signaling pathways of β5i knockout on EMT of podocytes, western blot was used to detect the EMT markers and cell signaling proteins. The results showed that β5i knockout significantly decreased the expression of α-SMA and fibronectin to 2.1 and 1.5 folds in STZ-treated β5iKO mice compared to 2.8 and 1.8 folds in STZ-treated C57/BL mice (*p* < 0.05, *n* = 6, Fig. 7e–g). Meanwhile, apelin significantly reversed the expression of α-SMA and fibronectin both in STZ-treated C57/BL and β5iKO mice (*p* < 0.05, *n* = 6, Fig. 7e–g).

### Effects of immunoproteasome β5i overexpression on TGFβ and NFκB pathway in podocytes

**Fig. 8:**
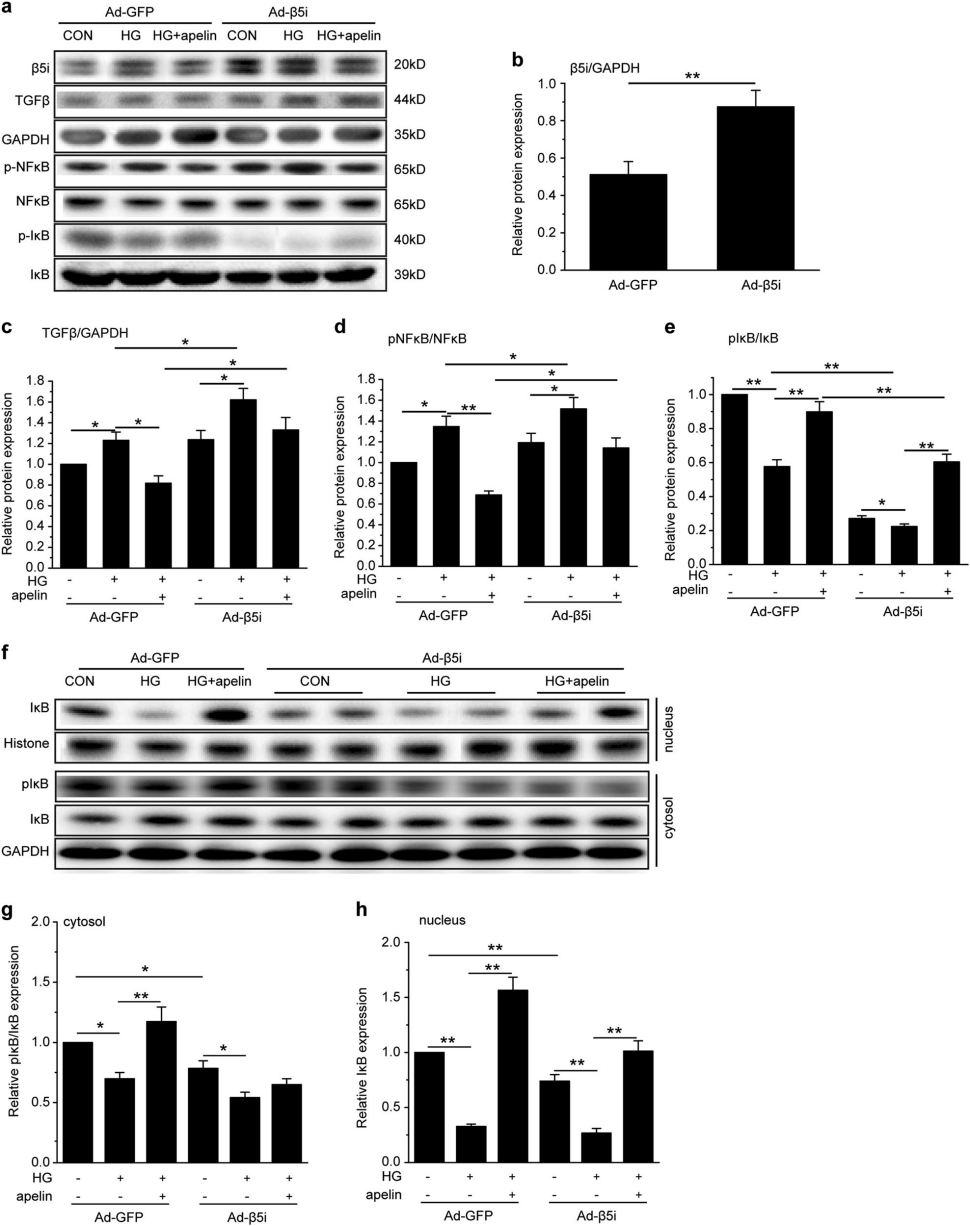
Proteasome subunit β5i overexpression canceled the inhibiting effects of apelin on cell signaling pathway of EMT in high-glucose-treated podocytes. **a** Representative images of western blot for cell signaling pathway in podocytes after proteasome subunit β5i overexpression using adenovirus. **b** Expression of proteasome subunit β5i were increased by adenovirus in podocytes (*n* = 3, **p < 0.01). **c** Overexpression of proteasome subunit β5i promoted expression of TGFβ in podocytes with or without apelin and/or high-glucose treatment. Values represent the mean ± SD (*n* = 3, *p < 0.05). **d** Overexpression of proteasome subunit β5i promoted phosphorylation of NFκB in podocytes with or without apelin and/or high-glucose treatment. Values represent the mean ± SD (*n* = 3, *p < 0.05, **p < 0.01). **e** Overexpression of proteasome subunit β5i inhibited phosphorylation of IκB in podocytes with or without apelin and/or high-glucose treatment. Values represent the mean ± SD (*n* = 3, *p < 0.05, **p < 0.01). **f** Representative images of western blot for IκB in cytoplasm and unclear of podocytes after proteasome subunit β5i overexpression using adenovirus. **g** Overexpression of proteasome subunit β5i inhibited phosphorylation of IκB in cytoplasm of podocytes with or without apelin and/or high-glucose treatment. Values represent the mean ± SD (*n* = 3, *p < 0.05, **p < 0.01). **h** Overexpression of proteasome subunit β5i decreased IκB in nucleus of podocytes with or without apelin and/or high-glucose treatment. Values represent the mean ± SD (*n* = 3, **p < 0.01)

To prove the effects of β5i on TGFβ and NFκB in podocytes, adenovirus was used to overexpress β5i in podocytes. The results showed that adenovirus infection significantly increased expression of β5i in podocytes to 1.78 folds (Fig. 8a, b). β5i overexpression significantly increased TGFβ in high-glucose-treated podocytes with or without apelin treatment (1.3 and 1.68 folds separately, *p* < 0.05, *n* = 3, Fig. 8a–c). Meanwhile, overexpression of β5i significantly increased the pNFκB in high-glucose-treated podocytes with or without apelin treatment (1.15 and 1.55 folds separately, *p* < 0.05, *n* = 3, Fig. 8a–d). However, the pIκB was significantly decreased to 0.24 folds by overexpression of β5i in high-glucose-treated podocytes, which was reversed by apelin to 0.59 folds (Fig. 8a–e). These results suggested that pIκB was degraded by proteasome. Therefore, IκB both in nucleus and cytosol were examined after β5i overexpression. The results indicated that pIκB in cytosol was decreased to 0.69 folds of control in high-glucose-treated podocytes and reversed to 1.19 folds in high-glucose and apelin-treated podocytes. Overexpression of β5i decreased pIκB in cytosol to 0.71 and 0.53 folds separately in high-glucose-treated podocytes with or without apelin treatment (Fig. 8f, g). IκB in nucleus was decreased to 0.32 folds of control in high-glucose-treated podocytes and reversed to 1.56 folds in high-glucose and apelin-treated podocytes. Overexpression of β5i decreased IκB in nucleus to 1.03 and 0.27 folds separately in high-glucose-treated podocytes with or without apelin treatment (Fig. 8f–h). These results suggested that β5i overexpression increased the degradation of pIκB in cytosol, and apelin promoted the translocation of IκB into nucleus.

## Discussion

In this study, the results highlighted the critical role of apelin on EMT of podocyte in DKD. Apelin, which was upregulated in diabetic mice (Fig. 1), alleviated glomerular fibrosis (as shown in Fig. 2) and decreased expression of EMT markers, such as α-SMA, collagen1, αfibronectin (Fig. 3), and TGFβ (Fig. 4) in diabetic mice. These effects were confirmed in cultured podocytes: apelin decreased expression of EMT markers (Fig. 5) in high-glucose-treated podocytes, which was correlated with expression of TGFβ/Smad and NFκB pathway (Fig. 6). The effects of apelin on EMT of podocytes were aggregated in STZ-treated β5iKO mice (Fig. 7) and canceled by overexpression of proteasome subunit β5i in podocytes (Fig. 8). These results suggested the inhibiting effects of apelin on EMT of podocytes in diabetic mice were dependent on decreased expression of β5i and the following downregulation of TGFβ/Smad pathway.

EMT of podocytes could be activated in diabetic conditions^15,16^ and resulted in glomerular fibrosis in ESRD^2^. Previous studies have demonstrated that TGFβ pathway and NFκB pathway played an important role in EMT of podocyte in diabetes mellitus^16,17^. However, it is still controversial about how to deal with EMT of podocyte by inhibiting these signaling pathways^18^. This study revealed that apelin inhibited TGFβ pathway and NFκB pathway by decreasing expression of β5i in podocytes.

First of all, the results showed that expression of apelin in glomeruli of diabetic mice was increased accompanied with glomerular fibrosis as shown in Figs. 1 and 2, which is consistent with previous reports^19^. This phenomenon could be explained by two ways related to podocyte EMT: it might be a compensatory reaction to suppress podocyte EMT induced by diabetes, or it might be a factor to induce podocyte EMT in DKD. Therefore, the effect of exogenous apelin on podocyte EMT in diabetic mice was detected. The results showed that apelin relieved the glomerular fibrosis and decreased the expression of EMT markers in glomeruli of diabetic mice, while F13A, the antagonist (or analog) of apelin, aggravated the glomerular fibrosis and expression of EMT markers in glomeruli of diabetic mice (Figs. 2 and 3). These results are similar with other published papers^6^^,^^20^, which suggested that apelin acted as a suppressing factor to relieve renal fibrosis by inhibiting EMT of podocytes.

To understand the mechanisms for apelin inhibiting EMT of podocyte during DKD, TGFβ/Smad and NFκB pathway, which were reported to be involved in EMT, were detected in both glomeruli and cultured podocytes. The results showed that both TGFβ/Smad and pNFκB were increased in diabetic conditions (in diabetic mice and high-glucose-treated podocytes) and reversed by apelin treatment (Figs. 4 and 6). Meanwhile, if BAY117082 was applied to decrease the activation of NFκB, expression and activation of TGFβ/Smad pathway were inhibited as well (Fig. 6g–i). These results indicated that activation of NFκB by apelin induced the expression of TGFβ and the following EMT of podocytes. Interestingly, proteasome subunit β5i was found to be increased in glomeruli of diabetic mice and high-glucose-treated podocytes, which was reversed by apelin treatment as well (Fig. 6). Investigators ever proposed that proteasome inhibitors might suppress transcription of TGFβ by regulating NFκB signaling pathways^13,19,21^. Therefore, it is hypothesized that proteasome subunit β5i might be the mediator for apelin to inhibit TGFβ through NFκB signaling pathway.

To confirm the effects of proteasome subunit β5i on apelin-suppressed EMT of podocytes in DKD, β5iKO mice were used to observe the effects of apelin on EMT of podocytes and glomerular fibrosis. The results showed that glomerular fibrosis and expression of α-SMA and fibronectin in podocytes of STZ-treated β5iKO mice were decreased compared to STZ-treated C57/BL mice, and apelin could not alleviate the glomerular fibrosis and expression of α-SMA and fibronectin in STZ-treated β5iKO mice (Fig. 7). Meanwhile, the TGFβ/Smad pathway was also decreased in β5iKO mice as well (Fig. 7). These results indicated the effects of apelin on EMT of podocyte were dependent on the expression of proteasome subunit β5i in podocytes.

To verify the effects of proteasome subunit β5i on TGFβ and NFκB signaling pathway in the EMT of podocytes, adenovirus were used to overexpress proteasome subunit β5i in podocytes. The results showed that overexpression of proteasome subunit β5i reversed the reduction of TGFβ induced by apelin in high-glucose-treated podocyte (Fig. 8a–c). At the same time, overexpression of proteasome subunit β5i increased pNFκB in high-glucose and apelin-treated podocytes, which seemed a paradox with decreased pIκB in the same condition (Fig. 8a–e).

It is reported that phosphorylated IκB could be degraded by proteasome to activate NFκB^22^, and proteasome inhibitor promoted translocation of IκB into the nucleus to combine with NFκB and suppress the transcription of TGFβ^13^. Therefore, the translocation of IκB into nucleus was detected in podocytes, the results showed that apelin increased the translocation of IκB into nucleus in high-glucose-treated podocytes, and overexpression of β5i canceled the translocation of IκB into nucleus (Fig. 8f–h) in high-glucose and apelin-treated podocytes. According to this point of view, proteasome subunit β5i might contribute to the inhibition of apelin on TGFβ/Smad pathway, which was mediated by translocation of IκB into the nucleus to inhibit the combination of NFκB with DNA in EMT of podocytes in diabetic conditions.

Even though apelin was reported to increase albuminuria in diabetic mice through increasing renal blood flow^23^ and inhibiting autophagy of pdocytes^24^, the present study demonstrated that EMT of podocytes in mice with DKD is suppressed by apelin, which was mediated by decreasing the transcription of TGFβ through inhibiting the expression of proteasome subunit β5i. Inhibition of β5i decreased the degradation of pIκB and induced translocation of IκB into nucleus to decrease the expression of TGFβ induced by NFκB. The present results support the hypothesis that decreased proteasome β5i involved in the inhibiting effects of apelin on EMT of podocytes by inducing translocation of IκB into nucleus to inactivate the protranscription effects of NFκB on TGFβ.

## Materials and methods

### Experimental animals

All animal studies followed the Animal Care and Use Committee of Capital Medical University (20100610). All animals received humane care, and the experimental protocol was approved by the Committee of Laboratory Animals according to the institutional guidelines.

Male kkAy mice and control C57BL/6J mice at the age of eight weeks were purchased from Capital Medical University (Beijing, China). Mice were housed in air-conditioned, specific pathogen-free animal quarters with lighting from 0800 to 2100 hours and were given unrestricted access to standard laboratory water throughout this study. Animals were fed on semi-purified moderately high-fat diet containing 24%kcal fat and 0.2% cholesterol.

The mice were randomly divided into saline group (C57 + saline group, *n* = 12, and kkAy + saline group, *n* = 12), which were intraperitoneally infused (using micro-osmotic pump form alzet, MODEL 1004, DURECT Corporation, Cupertino, CA95014, USA) with vehicle for 4 weeks; apelin treatment group (C57 + apelin group, *n* = 12, and kkAy + apelin, *n* = 12), which were intraperitoneally infused (using micro-osmotic pump form alzet, MODEL 1004, DURECT Corporation, Cupertino, CA95014, USA) with apelin-13 (A6469; Sigma-Aldrich, St. Louis, MO, USA, 30 μg/kg/day) for 4 weeks and F13A treatment group (C57 + F13A group, *n* = 12, kkAy + F13A, *n* = 12), which were intraperitoneally infused using micro-osmotic pump (alzet, MODEL 1004, DURECT Corporation, Cupertino, CA95014, USA) with F13A (the antagonist of apelin-13,057–29; Phoenix Pharmaceuticals, Strasbourg France, 25 μg/kg/day) for 4 weeks.

β5i knockout (β5iKO) mice on a C57BL/6 background were purchased from Jackson Laboratory (Bar Harbor, ME). All experiments were approved by the Animal Care and Use Committee of Capital Medical University and performed in accordance with the US National Institutes of Health Guide for the Care and Use of Laboratory Animals (publication no. 85e23, 1996). WT and β5iKO mice at 10–12 weeks of age were injected intraperitoneally with STZ (40 mg/kg/day)for 5 consecutive days^25^. The apelin was then intraperitoneally infused (using micro-osmotic pump form alzet, MODEL 1004, DURECT Corporation, Cupertino, CA95014, USA) for 4 weeks to observe the effects of β5i knockout on EMT of podocytes which was alleviated by apelin.

### Determination of apelin in kidney

The glomeruli were separated from the cortex of kidneys and homogenized by ultrasonic waves after the mice were sacrificed by overdosed anaesthetization. The supernatant was separated by centrifuging at 3500 × *g*, and aliquoted, stored at −80 °C, and assayed within 2 weeks.

Apelin concentrations were determined based on the competitive enzyme immunoassay principle with the mouse apelin C-terminus Enzyme Immunoassay Kit (EIAM-PC, RayBiothech, GA30092, USA). Briefly, biotinylated peptides were added simultaneously with the sample to the wells to competitively interact with the antibodies precoated on it, HRP-Streptavidin and color substrate were used to determine the biotin-peptides combined with the capture antibodies. The apelin concentrations were calculated according to the standard curve in each assay. The minimum detectable concentration of apelin is 5.84 pg/ml.

### Masson staining

Kidney of mice was fixed with 10% formalin and then sectioned at the coronal plane. The sections were deparaffinized and refixed in preheated Bouin’s Solution at 56 °C for 15 min. After removing the yellow color from sections with running tap water, these sections were stained in Biebrich Scarlet-Acid Fucshin for 5 min and in Phosphotungstic/Phosphomolydic Acid Solution for 5 min. Then the slices were stained with Aniline Blue Solution for 5 min to show the nucleus and mounted with xylene. The stained slices were scanned with a digital slide scanner (Pannoramic SCAN, 3DHISTECH, Budapest, HUNGARY) and the blue-colored area was quantified with ImageJ software to analyze the area of fibrosis.

### PicricSiriusred (PSR) staining

The slices were first immersed in Bouin’s fixative for 30 min and then washed with tap water, then the slices were immersed in Sirius Red solution (Direct Red 80 and saturated picric acid, Sigma) and briefly washed in 0.5% acetic acid (Thermo Fisher Scientific, Waltham, MA, USA). Finally the slices were mounted with a xylene-based mounting media (Richard-Allen Scientific, Kalamazoo, MI, USA). The photos were captured with a polarized microscope (80i, NIKON, Japan) and the collagen I and III were analyzed with ImageJ software.

### Immunostaining

Kidneys were embedded in OCT (4583, SAKURATissue-Tek&reg, Torrance, CA, USA) on dry ice. Five-micrometer sections were cut and performed with immunostaining. Briefly, the slices were fixed in 10% neutral buffered formalin; then washed with PBS and treated with 0.2% Triton X-100; mouse anti-αSMA (MS-113-P, Thermofisher, USA) and rabbit anti-synapotodin (sc-50459; Santa Cruz Biotechnology, Santa Cruz, CA, USA); or mouse anti-collagen 1α (ab6308, abcam, USA) and rabbit anti-synapotodin; or mouse anti-fibronectin (sc-271098, Santa Cruz, USA) and rabbit anti-synapotodin; or mouse anti-TGFβ (MAB1835, R&D, USA) and rabbit anti-synapotodin were incubated with the slices after blocked with 1% BSA; Donkey anti-mouse IgG-488 (ab150105; Abcam, Shanghai, China) and donkey anti-rabbit IgG-647 (ab150075; Abcam) were incubated with the slices; Hoechst 33342 was then used to stain the nucleus; the slices were mounted in VECTASHIELD Mounting Medium (H-1000, Vector Laboratories, Inc., Burlingame, CA, USA) staining. Images were obtained using a confocal microscope (TCS-SP8; Leica, Buffalo Grove, IL, USA). Positive cells with both synapotodin and EMT markers located in the glomeruli were counted and divided by synapotodin-positive cells located in the glomeruli. At least 100 glomeruli were analyzed in each group.

### Cell culture

Native podocytes were isolated from kidney of mice. Briefly, glomeruli were prepared by filtration of the cortex of kidney with mesh sieves, whose holes were100, 76, and 54 μm in diameter, then the tissues left on the mesh sieve with 54 μm holes were collected and transferred to cultural plates and incubated with 20% serum in mixture of DMEM and RPMI 1640 for 5 days. The cells were identified with synapotodin. Then the cells were used for the following experiments. After starvation with serum-free DMEM, the cells were modulated with BAY117082 (1.0 μmol/l) and/or apelin (1.0 nmol/l) or F13A (1.0 nmol/l) and/or treated with NG (5.5 mmol/l d-glucose) or HG (25 mmol/l d-glucose) for 7days. The cells were then collected for the detection of EMT markers. All the experiments were repeated for at least three times.

Conditionally immortalized mouse podocytes, kindly provided by P. Mundel (Mt. Sinai School of Medicine, New York, NY, USA), were cultured as previously described. When podocytes were well-differentiated, they were serum-starved overnight with serum-free RPMI 1640 medium. Then the cells were infected with adenovirus to overexpress proteasome subunit β5i. Then the cells were treated with NG (5.5 mmol/l d-glucose) or HG (25 mmol/l d-glucose) with or without apelin (1.0 nmol/l) modulation for 7days and collected for the cell signaling assays. All the experiments were repeated for at least three times.

### RNA extraction and qPCR

The separated glomeruli were homogenized with trizol (15596026, Invitrogen, Shanghai, China) and mixed with 200 µl of trichloromethane (Beijing Chemical Co., Ltd., Beijing, China). After centrifuging at 4 °C at 12,000 rpm for 15 min, the upper phase was treated with an equal volume (400 µl) of isopropanol (Beijing Chemical Co., Ltd., Beijing, China) at −20 °C for 2 h and then centrifuged at 4 °C at 12,000 rpm for 15 min to form a pellet. Subsequently, the pellet was washed with 75% ethanol and air-dried. The isolated total RNA was then re-dissolved in 20 µl diethylpyrocarbonate-treated (DEPC-treated) water (Shanghai Sangon Biology Technology and Services CO., LTD., Shanghai, China). Concentrations of the RNA samples were measured by detecting the optical density using a microplate reader (Eon, BioTek instruments, Inc. Winooski, Vermont 05404-0998 USA), and the purity was determined by the ratio of OD260 to OD280. The reverse transcription of first-strand cDNA was performed with a PrimeScript TMRT reagent kit (Takara, Nojihigashi, Japan) according to the manufacturer’s instructions. The qPCR was performed on a CFX96 Real-Time System instrument (Bio-Rad, Hercules, CA, USA) using SYBR Premix Ex Taq II (Takara, Nojihigashi, Japan). The cycling conditions were as follows: 95 °C for 30 s, 40 cycles of 95 °C for 5 s, and 60 °C for 30 s. All reactions were run in triplicate and were normalized to the reference gene *GAPDH*. All related primers were designed and synthesized by Shanghai Sangon Biology Technology and Services Co., Ltd. (Shanghai, China) and sequences were displayed in Supplementary Table 1.

### Extraction of nuclear and cytoplasmic protein

Nuclear and cytoplasmic proteins were extracted with an extraction kit (SC-003, Invent Biotechnologies, Inc. MN 55441, USA). Briefly, cells were washed with cold PBS for two times, cytoplasmic extraction buffer was added to lysis the cells on ice for 5 min after aspirating the PBS, and then transferred the cell lysate to a pre-chilled tube. Then the tube was vortex vigorously for 15 s and centrifuged for 5 min at 15,000 × *g* at 4 °C. The supernatant was the cytosol fraction and was transferred to a fresh pre-chilled tube. Nuclear extraction buffer was then added to lysis the pellet after washing with cold PBS for two times. The lysate of the pellet was then vortex vigorously for 15 s and incubated on ice for 1 min, which was repeated for four times. The nuclear extract was then immediately transferred to a pre-chilled filter cartridge with collection tube and centrifuged for 30 s at 15,000 × *g* at 4 °C. The lysate in the collection tube which was filtrated through the filter cartridge was the nuclear extract.

### Western blot analysis of protein expression

The proteins from glomeruli or cells were fractionated by electrophoresis on 10% SDS-PAGE, electroblotted to PVDF filter membranes, and incubated with the primary antibody at 4 °C and then with a horseradish peroxidase-conjugated secondary antibody. The experiment was repeated three times. Primary antibodies were mouse anti-collagen 1α (ab6308, abcam, USA), rabbit anti-αSMA (ab5694, abcam, Shanghai, China), mouse anti-fibronectin (sc-271098, Santa Cruz, USA), rabbit anti-TGFβ (ab92486, abcam, Shanghai, China), rabbit anti-Smad2/3 (8685 Cell Signaling Technology, Shanghai, China), rabbit anti-proteasome 20S LMP7 (β5i) (ab3329, abcam, Shanghai, China), rabbit anti-pIκB (2895S, Cell Signaling Technology, Shanghai, China), rabbit anti-IκB (4812, Cell Signaling Technology, Shanghai, China), rabbit anti-NFκB (8242, Cell Signaling Technology, Shanghai, China), rabbit anti-pNFκB (3033, Cell Signaling Technology, Shanghai, China), rabbit anti-histone H_3_ (9715, Cell Signaling Technology, Shanghai, China), rabbit anti-GAPDH (5174, Cell Signaling Technology, Shanghai, China). The antibody to glyceraldehyde 3-phosphate dehydrogenase (GAPDH) or β-actin was used to verify equal loading of proteins. Densitometry was performed with ImageJ software (National Institutes of Health, Bethesda, MD, USA).

### Statistics

Data are summarized as mean ± SD. A value of *p* < 0.05 was considered significant. All reported significance values are two-tailed. Analyses were performed with SPSS v13.0 for the PC (IBM, Armonk, NY, USA). Differences between groups were evaluated for significance with independent Student’s *t* test or one-way ANOVA and Newman−Keuls post hoc tests.

## Electronic supplementary material

supplementary table 1

supplemental figure 1
