# Supplementary material for: Apelin inhibited epithelial−mesenchymal transition of podocytes in diabetic mice through downregulating immunoproteasome subunits β5i
Source: Cell Death Dis. 2018 Oct 9;9(10):1031. doi: 10.1038/s41419-018-1098-4 (PMC6178343; doi:10.1038/s41419-018-1098-4)
Supplement: Supplementary file 1 — supplementary table 1 [file 41419_2018_1098_MOESM1_ESM.docx]

Supplementary table 1: primer sequences

| Target genes | Forward primer | Reverse primer |
| --- | --- | --- |
| Collagen1α | TAGGCCATTGTGTATGCAGC | ACATGTTCAGCTTTGTGGACC |
| Fibronectin | ATGCCTCGGGAATGGAAAGG | TCTAGCGGCATGAAGCACTC |
| α-SMA | ATCATCACCAACTGGGACGAC | TTTCTCCCGGTTGGCCTTAG |
| GAPDH | GGTTGTCTCCTGCGACTTCA | GGTGGTCCAGGGTTTCTTACTC |
