# Supplementary figures and images for: Apelin inhibited epithelial−mesenchymal transition of podocytes in diabetic mice through downregulating immunoproteasome subunits β5i
Source: Cell Death Dis. 2018 Oct 9;9(10):1031. doi: 10.1038/s41419-018-1098-4 (PMC6178343; doi:10.1038/s41419-018-1098-4)

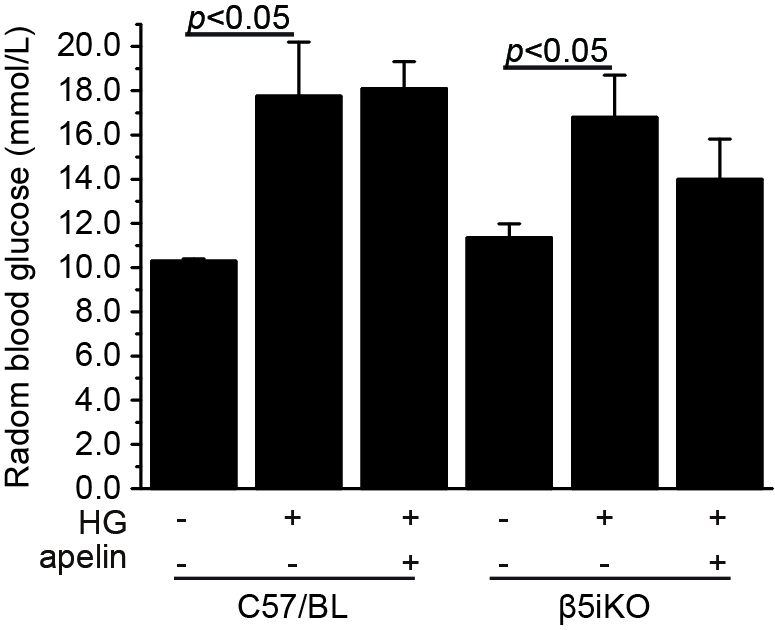

Supplement: Supplementary file 2 — supplemental figure 1 [file 41419_2018_1098_MOESM2_ESM.tif]
